# Unveiling the crucial role of ferroptosis in host resistance to streptococcus agalactiae infection

**DOI:** 10.1038/s41420-024-02189-8

**Published:** 2024-10-01

**Authors:** Jia-xuan Yi, Ze-yu Sun, Peng Liu, Yu-hang Wang, Hui Liu, Qing-yu Lv, De-cong Kong, Wen-hua Huang, Yu-hao Ren, Qian Li, Yong-qiang Jiang, Jing Li, Hua Jiang

**Affiliations:** 1 College of Biological Science and Food Engineering, Southwest Forestry University, Kunming, Yunnan China; 2 State Key Laboratory of Pathogen and Biosecurity, Academy of Military Medical Sciences, Beijing, China

**Keywords:** Microbiology, Cell death

## Abstract

IL-1β represents an important inflammatory factor involved in the host response against GBS infection. Prior research has suggested a potential involvement of IL-1β in the process of ferroptosis. However, the relationship between IL-1β and ferroptosis in the context of anti-GBS infection remains uncertain. This research demonstrates that the occurrence of ferroptosis is essential for the host’s defense against GBS infection in a mouse model of abdominal infection, with peritoneal macrophages identified as the primary cells undergoing ferroptosis. Further research indicates that IL-1β induces lipid oxidation in macrophages through the upregulation of pathways related to lipid oxidation. Concurrently, IL-1β is not only involved in the initiation of ferroptosis in macrophages, but its production is intricately linked to the onset of ferroptosis. Ultimately, we posit that ferroptosis acts as a crucial initiating factor in the host response to GBS infection, with IL-1β playing a significant role in the resistance to infection by serving as a key inducer of ferroptosis.

## Introduction

Group B Streptococcus (GBS) is a Gram-positive bacterium that can colonize the gastrointestinal and reproductive tracts asymptomatically. It acts as a primary pathogen in systemic infections in vulnerable individuals, particularly newborns and mothers, resulting in serious conditions, such as pneumonia, septicemia, and meningitis. GBS plays a significant role in the development of neonatal sepsis and meningitis.

IL-1β, which is an important mediator for the initiation and maintenance of inflammation and host defense, was reported to be significantly elevated in the early stages of GBS infection. Multiple studies showed that IL-1β has a strong stimulating effect on phagocytic cells, inducing the adhesion of neutrophils and monocytes to endothelial cells, and the stimulation of white blood cells and inflammatory factors such as lipid mediators and cytokines. *C. Biondo* et al. demonstrated the critical significance of IL-1β and IL-1R in host defense against GBS through their signaling pathways. IL-1β or its receptor deficiency can significantly reduce the production of the PMN chemokine CXCL1/2 and of the macrophage chemokine MIP-1β, limiting the effective recruitment of immune cells, which cause the host to be unable to effectively control the systemic spread of the bacterium to other target organs [1–4]. However, limited research exists regarding the relationship between IL-1β and macrophages in the host’s resistance to GBS infection.

Ferroptosis is a recently reported programmed cell death characterized by iron-dependent lipid peroxidation, which involves the accumulation of intracellular iron and lipid oxidation of cell membranes. Ferroportin (FPN) represents the sole exporter of intracellular iron, with a significant role in the regulation of plasma iron concentration. Additionally, while iron is exported to the plasma through FPN, hepcidin is a central regulatory factor in the systemic iron metabolism, being involved in the regulation of the transcription of FPN, while the iron itself enhances the binding of hepcidin to FPN [5]. In the presence of high extracellular iron concentrations, hepcidin binds to FPN, leading to FPN ubiquitination and degradation. Research suggests that loss-of-function mutations in FPN may be associated with heightened mortality rates in individuals diagnosed with bacteremia.

Research has indicated that IL-1β plays a regulatory role in ferroptosis-related molecules. *Tiziana P.* et al. reported that IL-1β can increase the excretion of iron ions from glial cells by inducing FPN and CP. Research from *MURIUKI J M*. has demonstrated that intestinal symbiotic bacteria have the ability to activate macrophages, resulting in the production of IL-1β. Subsequently, IL-1β triggers the upregulation of C/EBP-δ expression, thereby enhancing hepcidin expression through C/EBP binding. The elevated levels of hepcidin contribute to the development of inflammatory anemia [2, 6, 7]. Given the significant role of IL-1β in host defense against GBS and its strong association with cell ferroptosis, the objective of this study was to assess the potential relationship between the impact of IL-1β on host resistance to GBS infection and ferroptosis [8, 9].

## Results

### Ferroptosis plays a crucial role in the host’s resistance to GBS infection

**Fig. 1:**
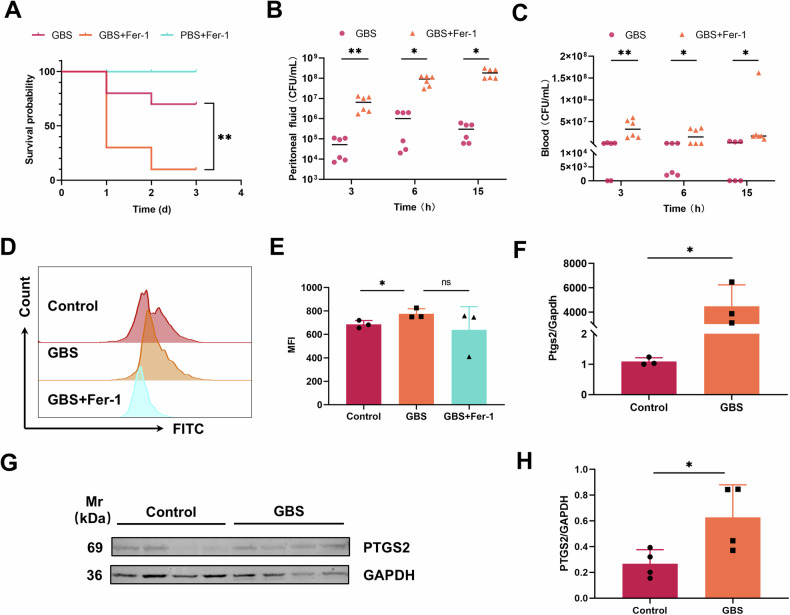
Ferroptosis promotes host resistance to GBS infection. **A** Mice were injected with 100 μL 0.2 mg/mL Fer-1 *i.p*. followed with infecting with 500 μL of 8×10^7^CFU/mL GBS *i.p*. Survival rate was observed (*n* = 10 per group). **B**, **C** Bacterial load was quantified in abdominal cavity and peripheral blood at 3, 6, and 15 hours post-infection. Each group included 6 animals. **D**, **E** Lipid oxidation levels were detected via flow cytometry after staining with Lipoflu at 3 hours postinfection (*n* = 3 per group). **F**–**H** The expression of PTGS2 was detected by qPCR(F), and Western blot and grayscale value analysis(G,H), at 3 hours post-infection(*n* = 4 per group). Data were shown as mean ± SD. *P < 0.05, **P < 0.01, ***P < 0.001, ns means not significant.

To examine whether ferroptosis enhances host resistance to GBS infection, the survival rate of infected mice was assessed through the administration of the ferroptosis inhibitor Fer-1. In this study, the survival rate of treated mice was less than 50%, resulting in a significant decrease in overall survival rates (Fig.1A). Simultaneously, peritoneal lavage fluid and peripheral blood samples were collected at various time points. The analysis indicated that bacterial counts were markedly elevated in the GBS+fer-1 group at 3, 6, and 15 hours post-infection, in comparison to the GBS-infected group, with the most pronounced disparity observed at 3 hours post-infection (Fig. 1B, C). These preliminarily results indicated that ferroptosis may be beneficial for the bacterial clearance in GBS-infected mice. During the host immune response, the oxidative stress is commonly induced to kill invading bacteria, while biochemical effects such as iron, ROS, and GSH also are responsible for substantial changes. We assessed the peritoneal lipid peroxidation using liperfluoro (Dojindo, Japan), and the Flow cytometry analysis proved that mice infected with GBS showed a lipid peroxidation, while after treatment with Fer-1, the lipid peroxidation was lower than that in the GBS infected group (Fig. 1D, E). The expression of PTGS2, a positive molecular marker of ferroptosis [10, 11], were significantly increased after 3 hours of infection from the peripheral lavage fluid, indicating that ferroptosis occurs in the host after GBS infection, playing a positive role in the resolution of infection (Fig. 1F–H).

Additionally, we seek to investigate whether enhancing host resistance to GBS infection can be achieved through preemptive and sustained induction of ferroptosis. Based on this, we used Erastin, which is a highly efficient stimulator of ferroptosis [8], however, after the Erastin treatment, we found that mice succumbed earlier after GBS infection(Supplementary Fig. 1A), suggesting that a moderate level of ferroptosis in the early stage of infection may play a crucial role in overcoming the infection, contrarily to a persistent or excessive ferroptosis that can weaken these effects.

Subsequently, in order to excluded the effects of other types of cell death on the immune response against GBS infection, we also evaluated the inhibitors 3-MA(3-methyladenine) and Nec-1 (Necrostatin-1), and found that they could also significantly inhibit the host resistance to GBS infection(Supplementary Fig. 1B). The relationship between autophagy and ferroptosis was extensively reported in the past [12–15] and studies conducted on the correlation between Nec-1 and lipid oxidation showed that Nec-1 can inhibit ferroptosis caused by sulfasalazine and Erastin (system xc-inhibitors) and Rsl3(a glutathione peroxidase 4 inhibitor) [14, 15]. In order to illustrate the inhibitory effect of Nec-1 here, we evaluated its effect on lipid oxidation after finding the ferroptosis stimulus. The results showed that Nec-1 could significantly inhibit lipid oxidation of macrophages(Supplementary Fig. 1C). It suggested that the function of Nec-1 here is at least related to its inhibition of lipid oxidation to some extent.

### The ferroptosis of macrophages is important for the host response to GBS infection

**Fig. 2:**
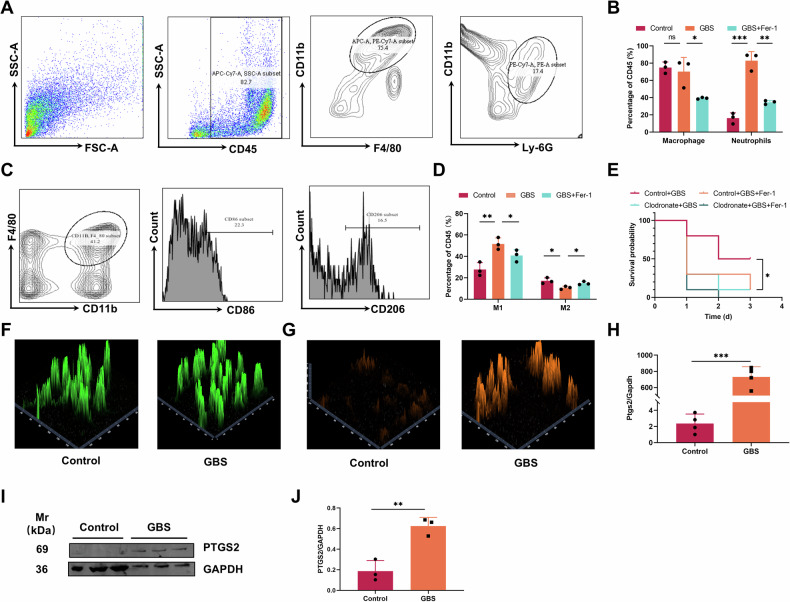
The ferroptosis of macrophages plays an important role for the host resistance to GBS infection. Mice were infected with 500 μL 8×10^7^ CFU/mL GBS *i.p*. Ratio of macrophages and neutrophils (**A**, **B**) and proportion of M1 and M2 macrophages (**C**,**D**) in peritoneal lavage fluid at 3 hours postinfection were assayed by flow cytometry. **E** Mice were treated with 200 μL clodronate lipome and control *i.p*. 24 hours before GBS infection and simultaneously Fer-1 treatment. The survival rates were compared. **F –J** Peritoneal macrophages from C57BL/6 mice were incubated with GBS at MOI of 1:30. The levels of lipid oxidation and Fe^2+^ were assessed using 100 μL of 2 μM C11-BODIPY^581/591^ and 200 μL of 1 μmol/L FerroOrange, respectively (**F**, **G**). The expression of PTGS2 was detected by qPCR (*n* = 4 per group) (**H**) and Western blot and grayscale value analysis (*n* = 3 per group) (**I**, **J**). Data are shown as mean ± SD. *P < 0.05, **P < 0.01, ***P < 0.001, ns means not significant.

The preliminary bacterial load and lipid oxidation results showed significant changes after 3 hours of infection, and we speculated that immune cells may also undergo significant changes during this time. After analyzing the peritoneal lavage fluid of infected mice, we found that the number of macrophages and neutrophils were significantly decreased after the inhibition of ferroptosis (Fig. 2A, B), indicating that the inhibition of ferroptosis reduced the host’s ability to overcome the infection. Macrophages are widely distributed in peripheral tissues, which can destroy invaded pathogens through phagocytosis and play an indispensable role in defending pathogens. Among them, M1 cells exhibit effective antibacterial properties, such as release of pro-inflammatory cytokines and stimulation of the Th1 response, while M2 cells support Th2-related effector functions [5, 16]. Through flow cytometry analysis, we found that the changes of M1 macrophages were the most obvious. Indeed, once the ferroptosis was inhibited, the proportion of M1 macrophages significantly decreased, while M2 macrophages significantly increased (Fig. 2C, D). Therefore, we investigated if macrophages were the main cell type responsible for ferroptosis. Using clodronate liposome to block macrophages in GBS-infected mice, we found that macrophage blockade significantly increased the mortality rate of infected mice (Fig. 2E). This rate was similar to the one observed in the Fer-1 treatment group, indicating that ferroptosis of macrophages plays an important role in the response to the infection. Subsequently, we investigated whether macrophages showed ferroptosis after infection. Peritoneal macrophages were isolated from mice and subsequently incubated with GBS at a ratio of 1:30 for a duration of 3 hours. The results showed that lipid oxidation of macrophages increased obviously after GBS infection (Fig. 2F). Previous literature reported that iron plays a crucial role in the immune response to bacterial infections, being also a key nutrient for bacterial survival in host cells [8]. We then investigated the intracellular ferrous content using FerroOrange incubation probe, and confocal imaging showed that after GBS infection, the intracellular ferrous fluorescence intensity and ferrous content increased (Fig. 2G), consistently with a significant increase in PTGS2 (Fig. 2H-J). All the above results showed that the ferroptosis of macrophages is crucial for the host to develop an efficient response to GBS infection.

### IL-1β may be the main reason for ferroptosis macrophages

**Fig. 3:**
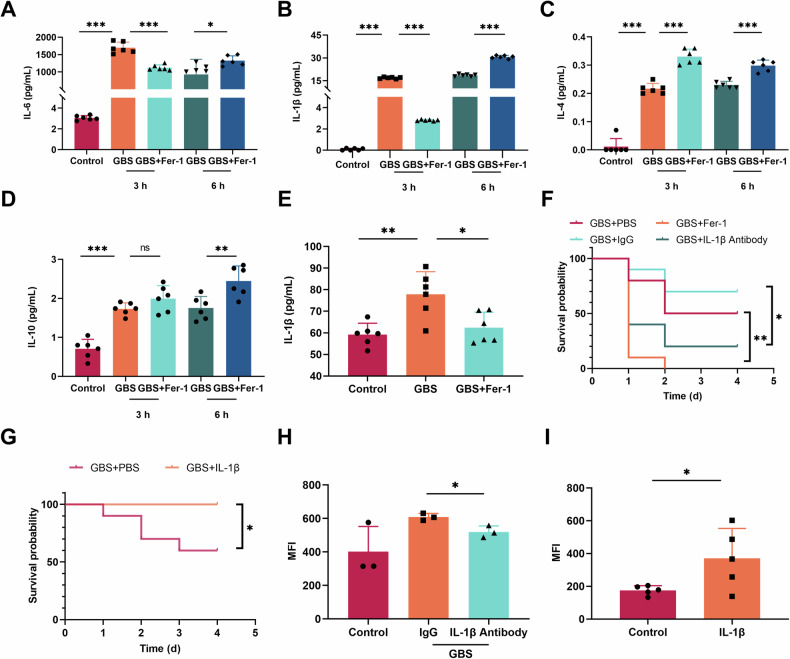
Association between IL-1β and ferroptosis macrophages. **A–D** Mice were infected with 500 μL 8 × 10^7^CFU/mL GBS *i.p*. Cytokines in peritoneal lavage fluid postinfection for 3 and 6 hours were detected by Luminex. **E** The level of IL-1β postinfection for 3 hours was detected by ELISA. **F** Mice were treated with 100 μL of 50 μg/mL mouse IL-1β/IL-1F2 antibody and homotypic control *i.p*. 0.5 hours before GBS infection and simultaneously Fer-1 treatment. The survival rate of mice was observed and compared by log-rank test (*n* = 10 per group). **G** Mice were injected with 500 μL of 100 pg/mL recombinant mouse IL-1β/IL-1F2(IL-1β) *i.p*. followed with GBS infection. The survival rate of mice was observed and compared by log-rank test (*n* = 10 per group). **H** IL-1β was blocked for 0.5 h followed by GBS infection for 0.5 h. Lipid oxidation level of peritoneal macrophages was detected by flow cytometry. **I** Mice were injected with 500 μL of 100 pg/mL IL-1β *i.p*.. Lipid oxidation levels of peritoneal macrophages were detected by flow cytometry. Data were shown as mean ± SD. *P < 0.05, **P < 0.01, ***P < *0.001,* ns means not significant.

Since ferroptosis is important for host resistance to GBS infection, we then investigated the stimuli that cause ferroptosis in the host. Multiple previous studies showed that IL-1β can promote host defense against GBS infection by recruiting immune cells to the infected site. In this regard, Luminex and ELISA results showed IL-1β at 3 hours after GBS infection, with IL-6, IL-4, and IL-10 significantly increased, however, IL-1β and IL-6 decreased significantly after treatment with Fer-1, while anti-inflammatory factors IL-4 and IL-10 significantly increased (Fig. 3A–E). At the same time, a significant increase in the number of bacteria was observed following the treatment with Fer-1 (Fig. 1B, C). It is suggested that the occurrence of ferroptosis in the early stage of infection can lead to the IL-1β production.

Given the critical role of IL-1β in mediating host resistance to bacterial infection [17], we subsequently implemented a blockade of the IL-1β produced by the host. Similar to Fer-1 treatment, the survival rate of mice in the IL-1β antibody blockade group significantly reduced (Fig. 3F). Furthermore, we injected IL-1β intraperitoneally (*i.p*.) into mice, and it was found that IL-1β could significantly promote host resistance to GBS infection (Fig. 3G). Meanwhile, following the IL-1β antibody blockade, the degree of lipid oxidation in peritoneal macrophages was found to be significantly decreased during GBS infection, but slightly higher than the background (Fig. 3H). On the other hand, when IL-1β was injected *i.p*., we found that in the absence of GBS infection, peritoneal macrophages showed a ferroptosis characterized by increased lipid oxidation (Fig. 3I), further indicating that IL-1β may be the main stimulus for macrophage ferroptosis.

**Fig. 4:**
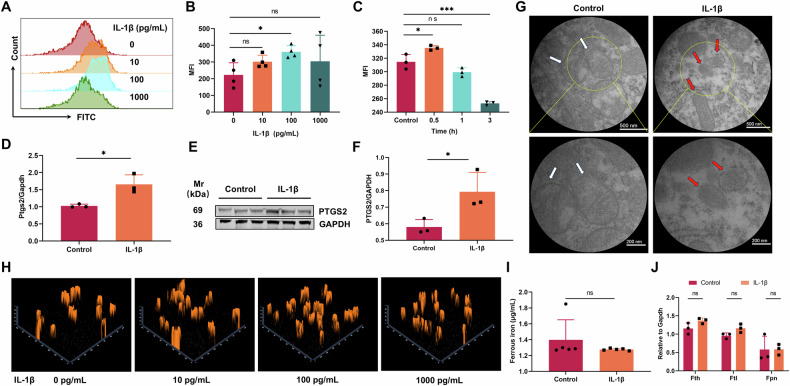
IL-1β caused ferroptosis of macrophages by direct lipid oxidation. **A–C** Macrophages were stimulated with 200 μL IL-1β at different concentrations (10 pg/mL, 100 pg/mL, and 1000 pg/mL) and different times (0.5 h, 1 h, 3 h). Lipid oxidation levels were analysed by flow cytometry. **D–G** Macrophages were stimulated with 200 μL of 100 pg/mL IL-1β for 0.5 hours. Expression of PTGS2 was evaluated by qPCR (**D**) and Western blot and grayscale value analysis (**E**, **F**). The mitochondrial morphology was observed by transmission electron microscopy (**G**). **H** Macrophages were stimulated with 200 μL IL-1β at different concentrations (10 pg/mL, 100 pg/mL, and 1000 pg/mL) for 0.5 hours and Fe^2+^ levels were detected via laser confocal fluorescence microscopy after staining with FerroOrange. **I**, **J** Macrophages were stimulated with 200 μL of 100 pg/mL IL-1β for 0.5 hours. Intracellular Fe^2+^ levels were evaluated by Iron Assay Kit (**I**) and the relative expression of Fth, Ftl and Fpn was evaluated by qPCR (**J**). Each group included 3-5 animals. Data were shown as mean ± SD. *P < 0.05, **P < 0.01, ***P < 0.001, ns means not significant.

Afterwards, different concentrations of IL-1β were used to stimulate RAW264.7, and we found that 100 pg/ml of IL-1β was able to significantly induce lipid oxidation in RAW264.7 (Fig. 4A, B), with a significant increase in RAW264.7 lipid oxidation at 0.5 hours (Fig. 4C). IL-1β stimulation also resulted in a significant upregulation of PTGS2 expression (Fig. 4D–F). Additionally, we observed characteristic morphological alterations in the mitochondria of IL-1β-treated RAW264.7 cells, including mitochondrial shrinkage, loss of cristae, and rupture of the outer mitochondrial membrane (Fig. 4G), which are consistent with the previously documented characteristics of ferroptosis [18].

Therefore, we hypothesized that IL-1β can be considered as the main stimulus for ferroptosis in macrophages during GBS infection. However, findings from previous luminex assays suggest that the production of IL-1β is also dependent on ferroptosis. Therefore, we investigated the cause of ferroptosis before its production. Multiple studies also demonstrated a link between bacterial infection and ferroptosis in host cells, with the result to decreased the host’s resistance to bacteria [19–21]. IL-1β is mainly produced by different type of cells during GBS infection, such as blood monocytes, tissue macrophages, and dendritic cells, while fibroblasts and epithelial cells usually are not involved in the production of cytokines [22]. Therefore, we investigated whether GBS infection can directly cause ferroptosis in macrophage. Interestingly, our results showed a significant induction of lipid oxidation and expression of Ptgs2 in RAW264.7 cells at 0.5 hours after infection when the MOI of GBS and RAW264.7 reached 10:1 or 30:1(Supplementary Fig. 2A, B), elucidating the observation of ferroptosis in peritoneal macrophages despite the inhibition of IL-1β. Previous studies have demonstrated that bacteria-induced ferroptosis is associated with an increase in intracellular iron ions [23]. However, in the present study, GBS infection did not result in a significant increase in intracellular iron content in RAW 264.7 cells(Supplementary Fig. 2C). This suggested that GBS might directly causes ferroptosis of macrophages or some other cells in the early stages of infection, which promotes IL-1β production. Although both GBS and IL-1β can cause ferroptosis in macrophages at the initial stage of infection, the ferroptosis caused by GBS may be relatively low, but it activates the release of IL-1β. As an inflammatory amplifier, IL-1β significantly activates the host’s natural immune system and promotes the host’s resistance to GBS infection.

### IL-1β causes macrophage ferroptosis by directly causing lipid oxidation

**Fig. 5:**
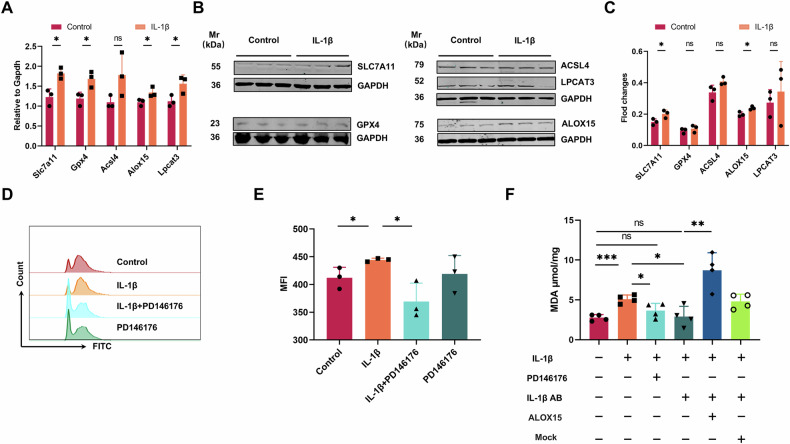
IL-1β induced lipid oxidation in macrophages through the upregulation of ALOX15 expression. **A–C** Macrophages were stimulated with 200 μL of 100 pg/mL IL-1β for 0.5 hours, and the expression levels of SLC7A11, GPX4, ACSL4, ALOX15, and LPCAT3 were detected by qPCR(A) and Western blot and grayscale value analysis (B,C). **D, E** Macrophages were treated with 100 μL of 1,000 μM PD146176 for 0.5 h, followed by IL-1β for 0.5 h, and flow cytometry was used to detect macrophage lipid oxidation levels. **F** Macropahges were incubated with 1000 μM PD146176 and 1 µg/mL mouse IL-1 β/IL-1F2 antibody for 0.5 hours, and then stimulated with 100 pg/mL IL-1 β for 0.5 hours. MDA levels were detected. Macropahges used here is RAW264.7, and RAW264.7 transfected with ALOX15(ALOX15 + ) or empty vector (Mock + ). The results were shown for three repetitions as the mean ± SD. Statistical significance: *P < 0.05, **P < 0.01, *P < 0.05. ns means not significant.

As the first line of defense against bacterial infections, macrophages developed several strategies for bacteria, such as limiting the iron acquisition, which is one of the most important regulatory pathway of host cells for iron homeostasis [24]. Considering the dependence of pathogenic microorganisms on iron, and it has been reported that IL-1β has a close relationship with iron transport by affecting the expression of hepcidin, FPN and CP [2, 25]. Is it thus likely that the excessive uptake of iron by cells is responsible for the macrophage ferroptosis? However, repeat experiments showed that, despite the constant observation of lipid oxidation in macrophages as well as the expression of PTGS2, there was no significant difference in the intracellular iron content between the IL-1β treatment and the control groups (Fig. 4H, I). Consistently, no significant changes were observed in the expression of key genes related to the metabolism of iron ions, such as Fth, Ftl and Fpn (Fig. 4J). Nonetheless, the results derived from qPCR and Western blot analyses revealed a significant elevation in the levels of SLC7A11, and ALOX15 following treatment with IL-1β. The expression of LPCAT3 exhibits variability at the nucleic acid level, while remaining consistent at the protein level. (Fig. 5A–C). The three genes aforementioned are closely related to lipid oxidation [26]. In detail, Slc7a11 is a key component of the system Xc, and the Xc-GSH-GPX4 axis is crucial for the cellular antioxidant activity, while ALOX15 and LPCAT3 are key genes inducing phospholipid oxidation and they can be both inhibited by GPX4 [26–28]. In order to determine the specific pathway responsible for lipid oxidation in the cell membrane, a model of mice with Slc7a11 knockout was developed(Supplementary Fig. 1D, E). Nevertheless, the findings from repeated experiments indicated that the deletion of Slc7a11 did not significantly affect the host’s capacity to combat GBS(Supplementary Fig. 1F). The changes in the number of immune cells showed that only peritoneal macrophages significantly decreased in Slc7a11^−/−^ mice after infection(Supplementary Fig. 1G). Nevertheless, abdominal macrophages derived from Slc7a11^−/−^ mice also exhibited substantial lipid oxidation following incubation with GBS, accompanied by a reduction in iron content(Supplementary Fig. 1H, I). According to the above results, Slc7a11 is not the main cause of lipid oxidation during GBS infection.

Consequently, our research shifted focus to evaluating the role of ALOX15 as a critical molecule in the functional activity of IL-1β. Notably, following the inhibition of ALOX15 expression using the inhibitor PD146176 [29], IL-1β failed to induce lipid oxidation in macrophages (Fig. 5D, E). Subsequently, we conducted a comparative analysis of IL-1β blockade and ALOX15 inhibition, revealing that both interventions significantly attenuate the incidence of lipid oxidation (Fig. 5F). Previous studies showed that ALOX15 overexpression in target cells can be achieved through transfection [30]. Notably, when ALOX15 is overexpressed in RAW264.7 cells(Supplementary Fig. 2D, E), a significant increase in lipid oxidation is observed even without IL-1β (Fig. 5F). Based on the aforementioned studies, we propose that IL-1β facilitates ferroptosis in macrophages through the upregulation of ALOX15 and the subsequent promotion of lipid oxidation.

## Discussion

**Fig. 6:**
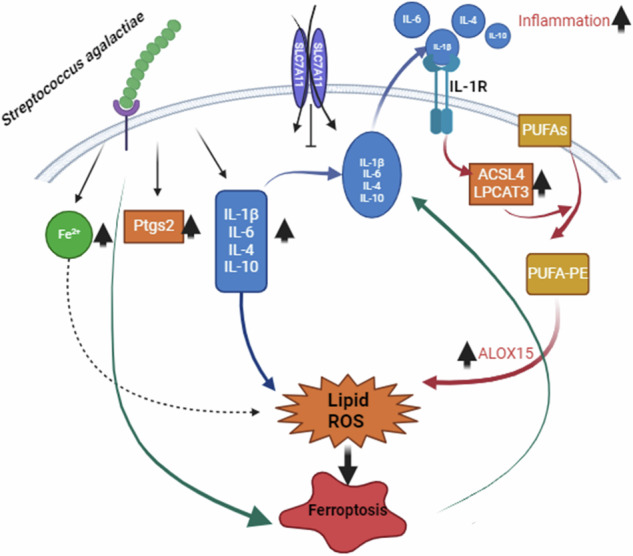
Diagram of a proposed model depicting the mechanism through which ferroptosis enhances host immunity against GBS infection. GBS infection induces macrophage ferroptosis involving iron accumulation and the release of inflammatory cytokines, including IL-1β. IL-1β is subsequently released into the extracellular space where it binds to its receptor, upregulating ACSL4, LPCAT3, and ALOX15. This process affected polyunsaturated fatty acid (PUFA) metabolism, ultimately leading to lipid oxidation and ferroptosis.

Ferroptosis was initially discovered in the study of cancer drugs, and several studies showed that it is closely related to many diseases, including infectious diseases. From this study it was suggested for the first time that a controlled of ferroptosis of macrophages in the early stages of infection plays an important role in the host’s resistance to GBS infection, while studies of stimuli have shown that IL-1β produced by the host in the early stage of infection is the main stimulus that causes ferroptosis in macrophages and thus stimulates the host anti-infection immune response. Previous studies have shown the important role of IL-1β for host resistance to GBS infection. Here, we propose that the anti-infectious effect of IL-1β on GBS can also liked by causing ferroptosis in macrophages (Fig. 6).

Many current studies on the relationship between ferroptosis and bacterial infections have shown that ferroptosis occurring in bacterial infections is harmful to the host [31]. To cite some examples, the induction of macrophage death during the infection sustained by *Mycobacterium tuberculosis* can promote the spread of the bacterium, while the Fer-1 inhibition of ferroptosis will significantly reduce the bacterial load [32], or the ferroptosis caused by *Staphylococcus aureus* infection showed that the bacterium causes a lipid membrane damage by interfering with the antioxidant mechanism of GPX4 [25]. However, in this study, when ferroptosis was inhibited by Fer-1, the mortality rate of GBS-infected mice significantly increased, indicating that ferroptosis plays an important role in the host’s resistance to GBS infection. However, when erastin was used to promote ferroptosis, the mortality was higher and occurred faster, even if in case of bacterial load is far from reaching the lethal dose. In summary, a moderate level of ferroptosis is crucial for the host resistance, but an excessive ferroptosis can also have counterproductive effects, impairing the host’s resistance to infection. Therefore, this led to our investigations on the difference between ferroptosis that occurs during GBS infection and ferroptosis promoted by erastin. As for the inflammation mediators, we observed that ferroptosis is accompanied by an increase of not only pro-inflammatory factors, but also of anti-inflammatory factors such as IL-4 and IL-10. This could be explained by the fact that the organism is able to balance the inflammation in the early stages of the infection caused by ferroptosis by initiating an anti-inflammatory response. In addition, the content of IL-1β at 3 hours and 6 hours post-infection did not significantly increased. Furthermore, our results from simultaneous colony counting of blood and peritoneal lavage fluid demonstrated a significant reduction in bacterial load at 6 hours post-infection. This suggests that, following effective control of the initial infection, IL-1β levels do not continue to rise, nor does ferroptosis further increase, which indicate that ferroptosis in the early stage of infection is consistently balanced in relation to the level of bacterial infection. As ferroptosis inducer, erastin is responsible for causing ferroptosis through multiple pathways [26] and since it is not regulated along with immune responses, it might lead to excessive ferroptosis, thereby weakening the immune response of mice, as well as higher mortality rates. As for which type of cells the production of anti-inflammatory factors is related to and whether it is related to the control of IL-1β production requires further study.

In the exploration of ferroptosis inducers, the initial consideration pertains to the potential of GBS to induce ferroptosis, for numerous investigations on ferroptosis and bacterial pathogenesis have demonstrated that bacteria can compromise host immunity by triggering iron-dependent cell death in host cells [12, 13]. Similarly, our results showed that GBS can also be responsible for macrophage ferroptosis, however, significant differences can only be observed when the MOI between GBS and cells reaches 500:1, which is not common in the early stages of infection, especially in less severe cases. When the MOI decreased to 30:1, we found that GBS exhibited also lipid oxidation in cells after incubation with RAW 264.7 for 30 minutes, suggesting a stimulating effect of GBS on macrophage lipid oxidation. However, further research is still needed on the underlying mechanism.

In addition to bacterial stimuli, the potential existence of host-derived stimuli is also being considered. Previous studies have highlighted the crucial role of IL-1β in host defense against GBS infection, with early production of IL-1β observed in both bloodstream and abdominal infections. Given the significance of IL-1β, our blocked experiments have confirmed its pivotal role as the primary stimulus for macrophage ferroptosis in the initial stages of infection. Hence, we suggest that IL-1β has the potential to not only attract immune cells through chemotaxis, but also stimulate the anti-infection immune response by inducing macrophage ferroptosis.

In light of the potential stimulus source of GBS, a comparison was made between ferroptosis triggered by IL-1β and by GBS. After treatment by IL-1β, macrophages were not completely damaged after lipid oxidation but still maintained a certain cellular morphology, and to a lesser extent also their functional activity. In contrast, macrophage death induced by GBS exhibited distinct cell rupture, which to some extent explains the regulatory effect of IL-1β on ferroptosis and subsequent inflammatory response.

Interleukin-1β(IL-1β) is a critical pro-inflammatory cytokine, and its secretion is well-documented to occur through the activation of the NOD-like receptor family pyrin domain containing 3(NLRP3) inflammasome and caspase-1. This pathway is implicated in various cell death mechanisms, including ferroptosis [33]. Studies demonstrated that both ferroptosis and pyroptosis involve the oxidation process, and that GPX4, as a lipid oxidoreductase, not only reduces lipids in ferroptosis, but also plays a role in the inhibition of the activation of inflammasomes and reduction of oxidized lipids in pyroptosis [34]. Mice with BMDM lacking GPX4 showed an increased susceptibility to polymicrobiota, however, the GPX4 deficiency did not affect the formation of inflammasomes [35], suggesting that the inflammasomes may occur prior to the ferroptosis. In this study, we hypothesize that IL-1β serves as a potential intermediary linking inflammasome activation to the onset of ferroptosis, a key factor in triggering the host immune response to GBS infection. Our findings suggest that ferroptosis may potentiate signals for inflammasome activation.

The inhibition experiments on Nec-1 and 3-MA showed a significant increase in mouse mortality rate. Furthermore, we found that Nec-1 can also inhibit lipid oxidation, indicating that Nec-1 may have off-target effects to some extent [15]. However, we cannot rule out that Nec-1 may also inhibit necroptosis, thereby affecting the production of inflammatory mediators. Several studies reported a close relationship between autophagy and ferroptosis, and therefore further research is needed about the role of autophagy in this regard [36, 37].

In conclusion, our study has identified a novel function of IL-1β in enhancing host defense against GBS infection by inducing ferroptosis in macrophages. This process involves the initiation of host resistance to GBS infection through the direct mechanism of lipid oxidation of the cell membrane via the LPCAT-ALOX15 pathway, rather than by increasing the cellular iron ion concentration [26]. Our proposition suggests that pathogens have the ability to hinder the host’s defense response by inducing ferroptosis, while the host can potentially combat pathogen infection by modulating ferroptosis. The extent of regulation of ferroptosis plays a crucial role in determining the host’s ability to resist infection. Further investigation is required to understand the mechanisms by which the host regulates the occurrence of ferroptosis and the prompt termination of this process following infection control. Additionally, our observations of phenomena associated with other forms of cell death warrant further exploration.

## Materials and methods

### Ethics statement

This research was conducted in compliance with the guidelines of laboratory animal care approved in China. All experiments and procedures were approved by the animal ethics committee of the Academy of Military Medical Sciences.

### Bacterial strains

The GBS strain BJCH_SA4 used here is a high virulent type III strain which is stored in our lab. The bacteria were grown overnight in Todd–Hewitt broth(THB) at 37 °C containing 5% CO_2_, washed twice in nonpyrogenic PBS after centrifugation(PBS; 0.01 M phosphate, 0.15 M NaCl, pH 7.2), and resuspended to the desired concentration.

### Animals

Six-eight-week specific pathogen-free male C57BL/6 J mice were purchased from Charles River(Beijing, China). Slc7a11^−/−^mice were constructed by Cyagen Biosciences, Guangzhou, China. The animals were housed and bred under pathogen-free conditions within the animal facilities of the Academy of Military Medical Sciences.

### Experimental models of GBS disease

Six-week-old mice, randomly divided into two group respectively, injected *i.p*. with 0.5 ml 8 × 10^7^ CFU/mL GBS and were subsequently monitored daily for survival. Following infection, peripheral blood was collected from the orbital vein into an EDTA anticoagulation tube. Subsequently, saponin was added, and the sample was lysed on ice for 5 minutes. The lysate was then diluted to the appropriate concentration and spread onto a THB agar plate. The plates were incubated overnight at 37 °C to facilitate colony enumeration.

### Cytokine measurement

Cytokine concentrations in peritoneal lavage fluid were quantified using a multiplexed Luminex xMAP assay(Cytokine & Chemokine 36-Plex Mouse ProcartaPlex™ Panel 1 A, Thermo Scientific, USA) or ELISA according to the manufacturer’s instructions.

### Flow cytometry

Following a 3-hour infection period, peritoneal lavage fluid was collected into a tube. Red blood cells were lysed on ice for three minutes and subsequently centrifuged at 2300 rpm for three minutes. The supernatant was then removed to isolate the cells. The absolute count of peritoneal immune cells were determined by BD Celesta(BD Biosciences) and data were analyzed using FlowJo software. The fluorescent antibodies utilized here were as follows: TruStain FcX™PLUS(anti-mouse CD16/32)(156604), APC/Cyanine7 anti-mouse CD45(103116), PerCP/Cyanine5.5 anti-mouse F4/80(123125), FITC anti-mouse Ly-6G(127606), APC anti-mouse Ly-6C(128016), PE anti-mouse CD86(159203) and APC anti-mouse CD206(MMR)(141707) were purchased from BioLegend. PE-CF594 Rat Anti-CD11b(562317), Fixable Viability Stain 510(564406) and Fixable Viability Stain 700(564997) were purchased from BD Horizon™.

### Isolation and stimulation of macrophages

To isolate peritoneal macrophages, the peritoneal cavity of C57BL/6 J mice should be gently washed with 4 mL of ice-cold DMEM. The recovered buffer should then be subjected to centrifugation at 400 g for 10 minutes. Following centrifugation, the cell pellet should be resuspended and subsequently seeded into a cell culture flask containing DMEM supplemented with 10% FBS and allowed to adhere overnight at 37 °C in a 5% CO_2_ atmosphere.

For stimulation, RAW264.7 cells were seeded in 24-well plates at a density of 1 × 10^5^ cells per well in DMEM supplemented with 10% FBS. The cells were incubated overnight at 37 °C in a humidified atmosphere containing 5% CO_2_. On the following day, the medium was aspirated, and the cells were washed twice with DMEM. Subsequently, the RAW264.7 cells were stimulated with 1000 μM PD146176(MedChemExpress) and 1 μg/mL mouse IL-1β/IL-1F2 antibody(BioTechne) for 0.5 hours, followed by treatment with 100 pg/mL recombinant mouse IL-1β/IL-1F2(BioTechne) for an additional 0.5 hours.

### RAW264.7 transfection

Cells were seeded onto a 6-well plate at a density of 4 ×10^5^ cells per well. Transfection was performed when the cells reached 60% to 80% confluence, utilizing jetOPTIMUS(Polyplus-transfection®) to deliver 5 µg of plasmid DNA per well(mAlox15 pCDNA3.1-3xFLAG-T2A-EGFP for ALOX15 and pCDNA3.1-3xFLAG-T2A-EGFP as control, Youbio Biological Technology Co., Ltd.). Twenty-four hours post-transfection, the medium was replaced with DMEM supplemented with 10% FBS. Subsequently, at 48 hours post-transfection, the cells were washed with DMEM medium prior to further processing.

### Western Blot

RAW264.7 cells were harvested following washing with ice-cold PBS, and lysed in RIPA buffer(Beyotime Biotechnology) supplemented with protease inhibitor cocktails(1%, Sigma-Aldrich). The lysate was subjected to centrifugation at 12,000 g for 10 mins at 4 °C. Subsequently, protein concentrations were quantified and calibrated utilizing a BCA Protein Assay Kit(Beyotime Biotechnology). Proteins were separated through SDS-PAGE and subsequently transferred to PVDF membranes. After being blocked for one hour in 0.05% TBST and 5% skimmed milk. The membranes were then washed 3 times with TBST for 5 min each, followed by incubation with specific primary antibodies at 4 °C overnight and secondary antibodies at room temperature for 1 hour. Secondary antibodies were IRDye 680RD Goat-Mouse(LI-COR Biosciences, 926-68070) and IRDye 800CW Goat-Rabbit(LI-COR Biosciences, 926-32211). An Odyssey Infrared Imaging system(LI-COR Biosciences) was used to scan the immunoblots. Western blot images from the Odyssey imager were acquired as grayscale files for quantification with ImageJ software.

Primary antibodies were diluted in TBST containing 5% BSA. The following primary antibodies were utilized: PTGS2 polyclonal antibody(1:500, Proteintech Group, 27308-1-AP), GPX4 monoclonal antibody(1:1,000, Proteintech Group, 67763-1-Ig), ACSL4 polyclonal antibody(1:2,000, Proteintech Group, 22401-1-AP), LPCAT3 monoclonal antibody(1:1,000, Proteintech Group, 67882-1-Ig), GAPDH monoclonal antibody(1:5,000, Proteintech Group, 60004-1-Ig), ALOX15 Rabbit pAb(1:500, Beijing Biosynthesis Biotechnology, bs-24356R) and CCBR1 Rabbit pAb(1:500, Beijing Biosynthesis Biotechnology, bs-6883R).

### Transmission electron microscopy (TEM)

The mitochondrial structure was examined by standard transmission electron microscopy. Cells were fixed with 2.5% glutaraldehyde overnight, washed, dehydrated, and embedded in resin according to standard procedures. Mitochondrial structure were analyzed by a TEM(HT7800, Hitachi Ltd).

### MDA detection

The cells were seeded in a 6-well plate at a density of 1 × 10^6^ cells per well. Cell lysis was performed utilizing Western and IP cell lysis buffer. The protein concentration was subsequently quantified employing a BCA protein assay kit. The supernatant obtained from the lysate was then analyzed for MDA levels using a commercial assay kit. The testing is strictly carried out according to the instructions of kit. Western and IP cell lysate, MDA assay kit, and BCA protein concentration assay kit were all purchased from Beyotime in China.

### qPCR

Total RNA was isolated by RNeasy Kit(Qiagen, Germantown, MD, USA) with DNAse treatment according to the manufacturer’s instructions. RNA concentration was measured by a Nanodrop 2000 spectrophotometer(Thermo Fisher, USA). Reverse transcription(RT) was performed using a cDNA Synthesis Supermix(Novoprotein, USA). The resulting cDNA was subjected to real-time PCR with the SYBR qPCR Supermix Plus(Novoprotein, USA). Relative mRNA levels were calculated with the 2^-△△^Ct method using GAPDH as an internal control. Reactions were performed in triplicate with at least three biologic replicates. Primer sequences are shown in Table 1.

**Table 1:** The list of primers in this study.

	Gene	Primers
F1	FTH	5’-GCGTCTCCCTCGCAAGTG-3’
R1	FTH	5’-GGCATACAACTCCAGGTTGATCT-3’
F1	FTL	5’-CTGCATGCCCTGGGTTCT-3’
R1	FTL	5’-GATAGTGGCTTTCCAGGAAGTCA-3’
F1	FPN	5’-TCTTGTGTGTGATCTCCGTATTCA-3’
R1	FPN	5’-GAACGGATATCTTCAAATGGAGAAA-3’
F1	GAPDH	5’-CATGGCCTTCCGTGTTCCTA-3’
R1	GAPDH	5’-GCGGCACGTCAGATCCA-3’
F1	LPCAT3	5’-GGATGCCTGTGCCAACATG-3’
R1	LPCAT3	5’-GGCGATGGTGCCATTGA-3’
F1	ALOX15	5’-TCAGGCTTGCCACTTCATCAC-3’
R1	ALOX15	5’-CTGGCCAAGATGGATGGAA-3’
F1	ACSL4	5’-CCAAAGAACACCATTGCCATT-3’
R1	ACSL4	5’-AAGTCTGTGCTGCAATCATCCA-3’
F1	SLC7A11	5’-CGGATCAGGCATCTTCATCTC-3’
R1	SLC7A11	5’-CCACGCTGCCCGTGTT-3’
F1	GPX4	5’-GCTGTGCGCGCTCCAT-3’
R1	GPX4	5’-CCATGTGCCCGTCGATGT-3’
F1	PTGS2	5’-CAGCCAGGCAGCAAATCC-3’
R1	PTGS2	5’-ACATTCCCCACGGTTTTGAC-3’

Primers for Slc7a11-KO PCR.

F1: 5’-TACTGTGATAAGCAGTGTAGAGGG-3’.

R1: 5’-GTTATAGGAAATGGCCGTCAGATG-3’.

Product size: 513 bp.

Primers for Slc7a11-WT PCR.

F1: 5’-TACTGTGATAAGCAGTGTAGAGGG-3’.

R2: 5’-AAGGGGATGCTTTGCTATTCTCTA-3’.

Product size: 300 bp.

### Laser scanning confocal microscopy

Lipid peroxidation and intracellular Fe^2+^ levels were quantified using C11-BODIPY^581/591^ and FerroOrange, respectively, via laser scanning confocal microscopy(LSCM, Carl Zeiss, Germany). Pretreated cells(5 × 10^5^) were incubated with C11-BODIPY^581/591^ working buffer(2 μM) at 5% CO_2_, 37 °C in a homothermal incubator for 30 min and then observed by LSCM. Similarly, stimulated cells(5 × 10^5^) were stained with a FerroOrange probe at the concentration of 1 μM followed by incubated at 5% CO_2_, 37 °C in a homothermal incubator for 30 min and then detected by LSCM.

### Identification of genotype in Slc7a11^−/−^ mice

Tail tissue (18–20 g) from mice was collected and placed in a sterile 1.5 ml tube. DNA was extracted using Mouse quick genotyping kit(Beijing YangGuangBio life science) followed by PCR amplification. The amplified DNA was then run on a 1% agarose gel for electrophoresis to determine the mouse genotype.

### Reagents

Fluorescent dye used to detect ferroptosis, including C11-BODIPY^581/591^ was purchased from Invitrogen, Carlsbad, CA, while FerroOrange probes(F374) and Lipoflu were obtained from DojinDo, Tokyo, Japan. Iron Assay Kit were obtained from Sigma, USA. ELISA kits of IL-1β was supplied by Biosource, Worcester, MA. Erastin (Era) (S7242), Fer-1(S724302), and 3-MA(S276713) Nec-1(S803703) were purchased from Selleckchem, USA.

### Statistical Analysis

All data were analyzed with GraphPad Prism (version 8.4.2, GraphPad Software, La Jolla, CA, USA). The differences between groups were calculated using an unpaired t-test and a log-rank test, respectively. Data from parameters were evaluated and indicated as mean ± standard deviation (SD) for two or three reduplicative experiments. Differences were considered statistically significant when *P* < 0.05: *P < 0.05, **P < 0.01, and ***P < 0.001.

## Supplementary information

Supplementary information

Original Data

## Data Availability

The datasets used and analyzed are available upon request from the corresponding author.
