# Supplementary material for: Unveiling the crucial role of ferroptosis in host resistance to streptococcus agalactiae infection
Source: Cell Death Discov. 2024 Oct 1;10:423. doi: 10.1038/s41420-024-02189-8 (PMC11445261; doi:10.1038/s41420-024-02189-8)
Supplement: Supplementary file 1 — Supplementary information [file 41420_2024_2189_MOESM1_ESM.pdf]

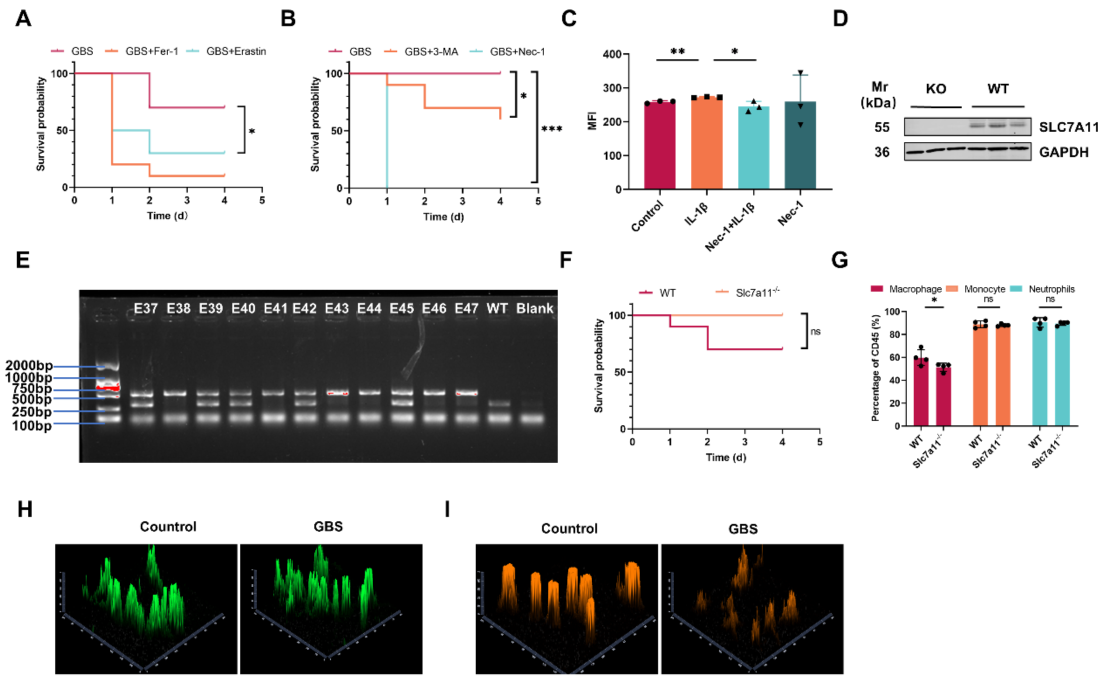

Supplementary Fig. 1 A,B Mice were intraperitoneally injected with 100 µL of 4 mg/mL Erastin, 0.2 mg/mL Fer-1, 0.2 mg/mL Nec-1, or 4 mg/mL 3-MA, followed by an intraperitoneal injection of 500 µL of GBS at a concentration of 8 × 10^7 CFU/mL. The survival rate was subsequently monitored(n=10 per group). **C** RAW264.7 was stimulated with 100µL 100µM Nec-1 and 200µL 100 pg/mL IL-1β for 0.5 hours and flow cytometry was used to detect lipid oxidation levels. **D,E** Slc7a11 knockout mice detection by WB(D) and PCR(E). Slc7a11$^{-/-}$ mouse PCR product identification criteria: Homozygotes: one band with 513 bp; Heterozygotes: two bands with 513 bp and 300 bp; Wildtype allele: one band with 300 bp. **F** Survival rate of mice infected by GBS(n=10 per group). **G** Proportion of peritoneal immune cells of WT and Slc7a11$^{-/-}$ mice infected for 3 hours was detected by flow cytometry. **H,I** Peritoneal macrophages were collected from Slc7a11$^{-/-}$ mice and incubated with GBS at a MOI of 1:30. Lipid oxidation and Fe^{2+} were detected using 200 µL 1µmol/L of FerroOrange, and 100 µL 2µM C11-BODIPY$^{581/591}$ respectively by laser confocal fluorescence microscopy. Data from three independent experiments were presented as the mean ± SD (n=3 per group). Statistical significance: *$*P<0.05$, $**P<0.01$*; *$*P<0.05$*; ns means not significant . The survival rates of the two groups were compared using a log-rank test.

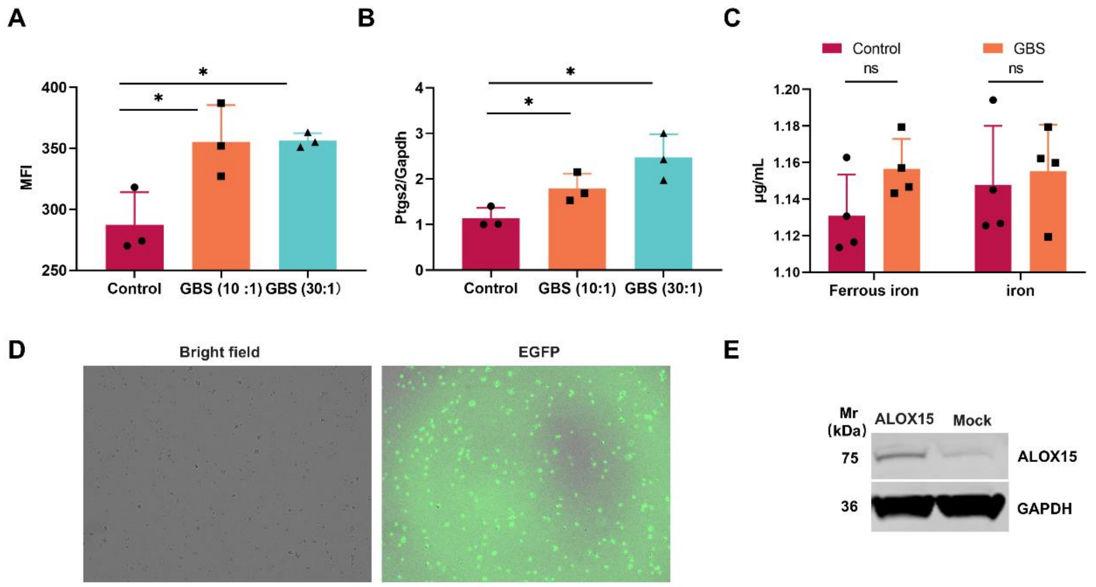

Supplementary Fig. 2 RAW264.7 cells were incubated with GBS for 0.5 hours at MOI of 10:1 and 30:1. Lipid oxidation levels and the expression of Ptgs2 were detected by flow cytometry(**A**) and qPCR(**B**) respectively. **C** Total iron and ferrous were detected by Iron Assay Kit. **D,E** Transfection efficiency(D) and expression of ALOX15(E) in RAW264.7. Data were presented as the mean ± SD from three replicates. Statistical significance: **P<0.05, **P<0.01, ***P<0.001* vs. the control group; ns means not significant.
