# Supplementary material for: Unveiling the crucial role of ferroptosis in host resistance to streptococcus agalactiae infection
Source: Cell Death Discov. 2024 Oct 1;10:423. doi: 10.1038/s41420-024-02189-8 (PMC11445261; doi:10.1038/s41420-024-02189-8)
Supplement: Supplementary file 2 — Original Data [file 41420_2024_2189_MOESM2_ESM.pdf]

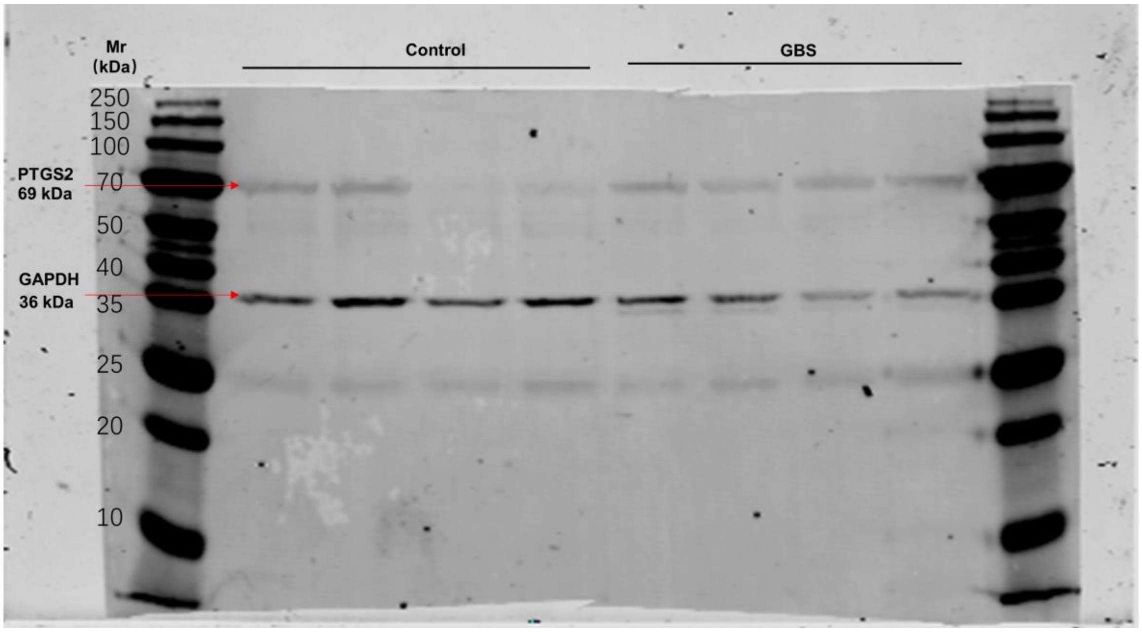

Uncropped western blotting membranes of Fig.1G.

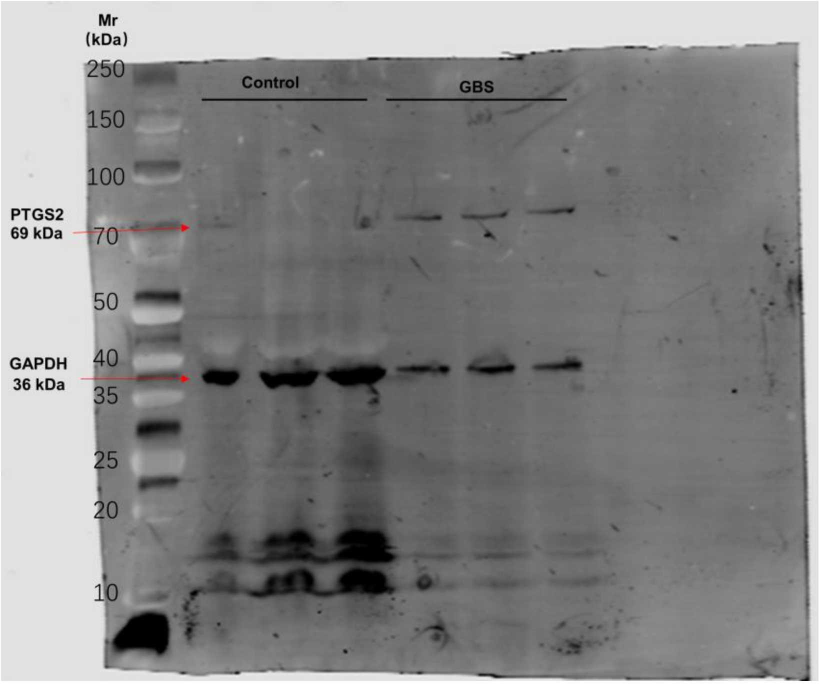

Uncropped western blotting membranes of Fig.2I.

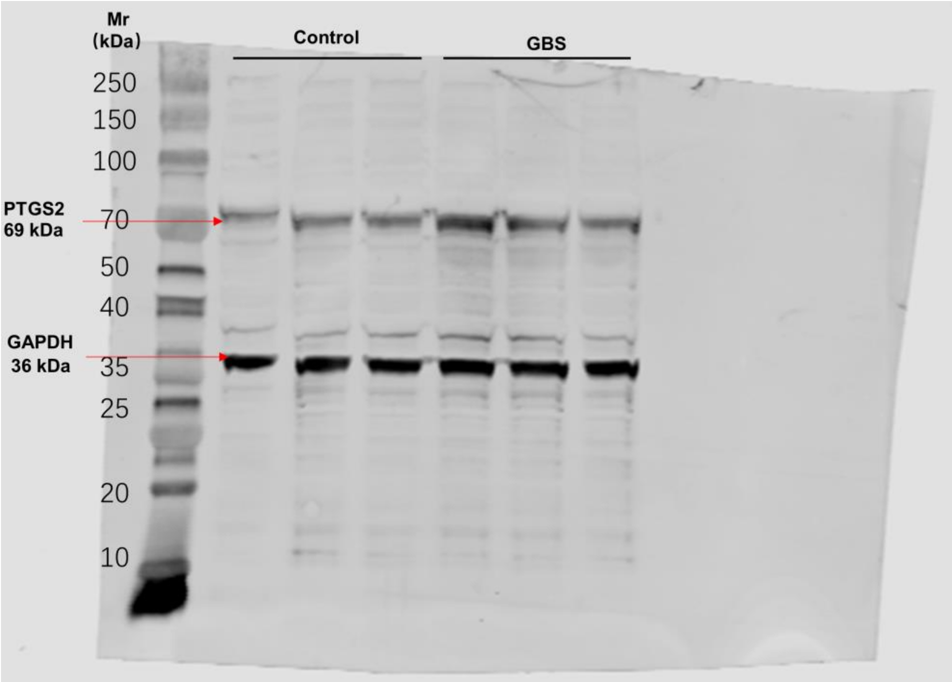

Uncropped western blotting membranes of Fig.4E.

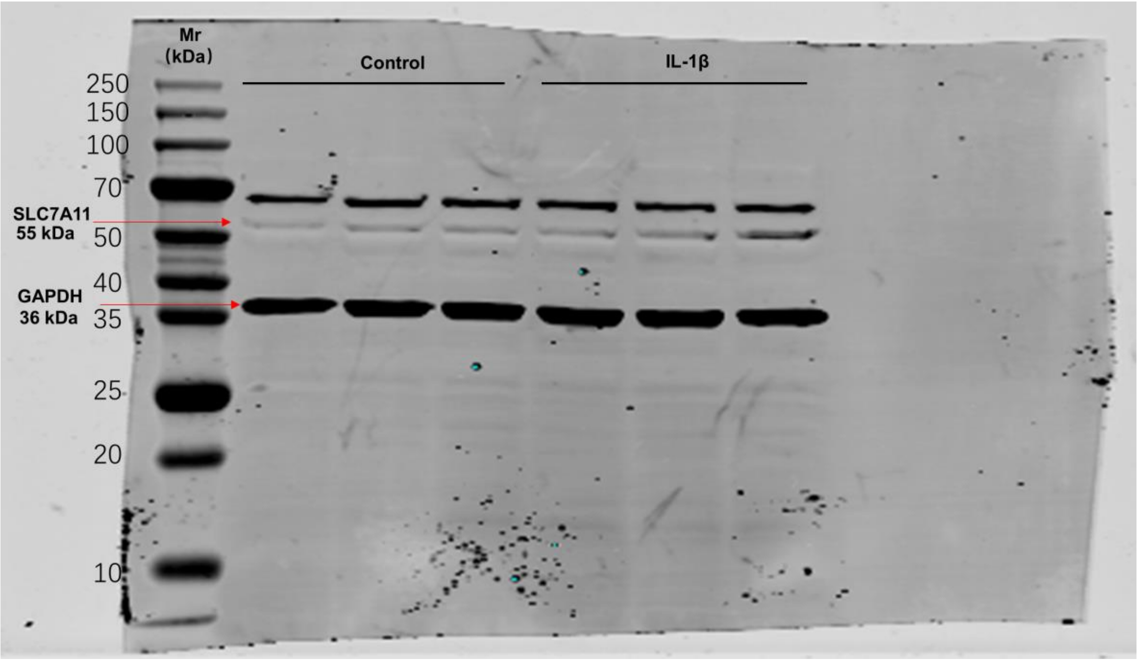

Uncropped western blotting membranes of Fig.5B（a）.

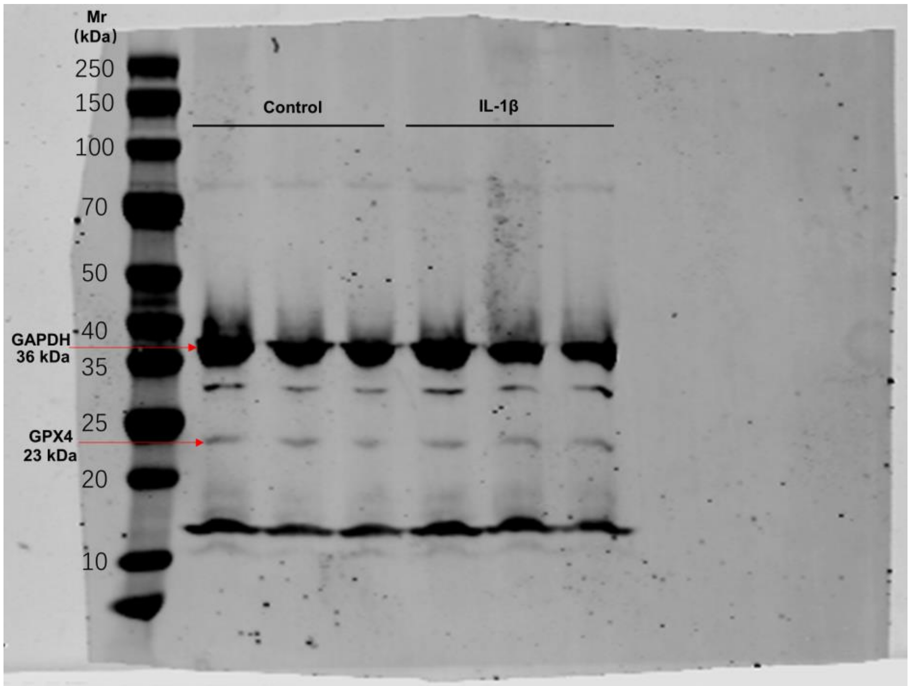

Uncropped western blotting membranes of Fig.5B（b）.

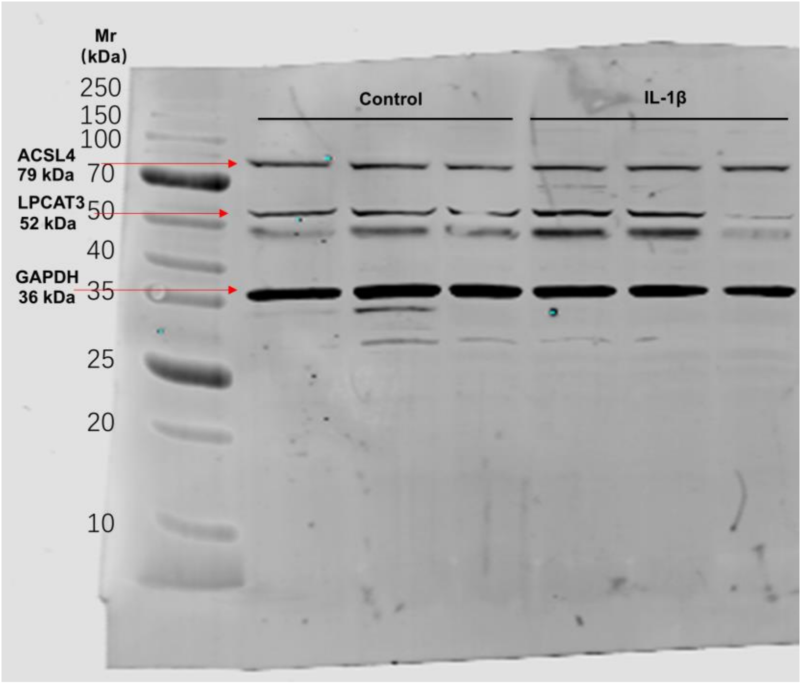

Uncropped western blotting membranes of Fig.5B（c）.

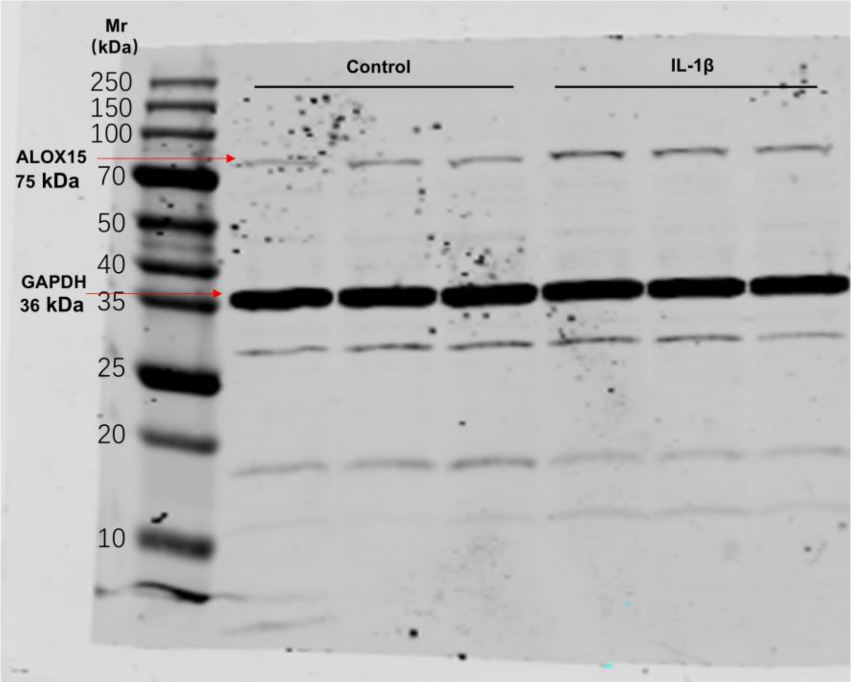

Uncropped western blotting membranes of Fig.5B（d）.

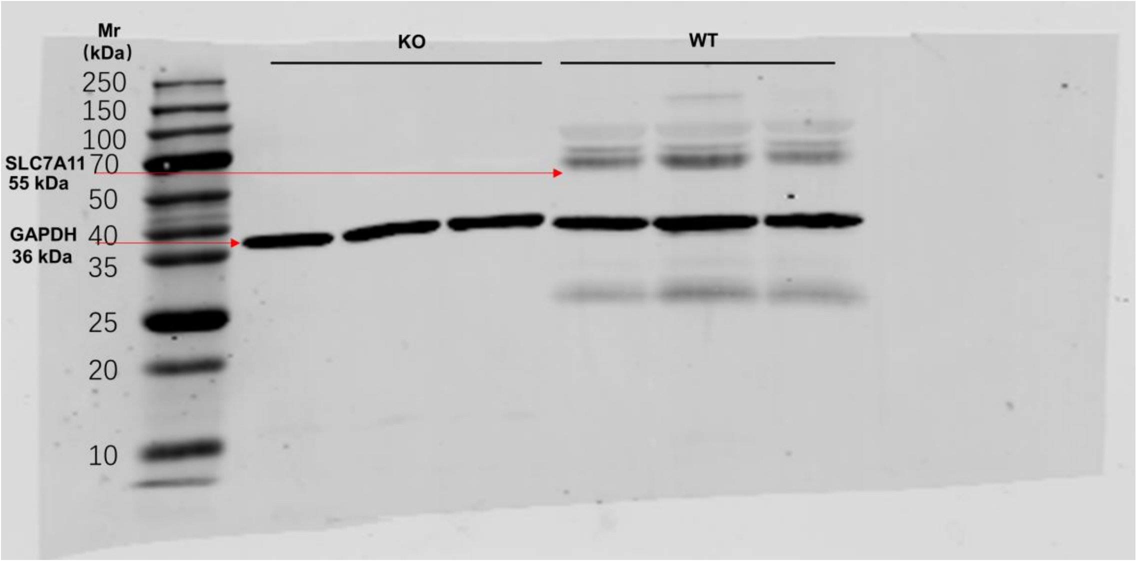

Uncropped western blotting membranes of Supplementary Fig. 1D.

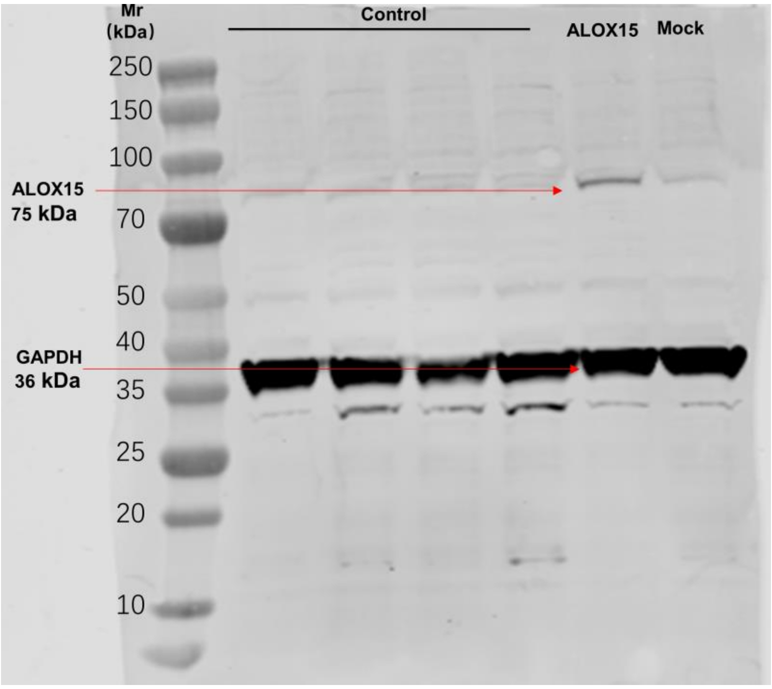

Uncropped western blotting membranes of Supplementary Fig. 2E.
